# Supplementary material for: Dynamic transcriptomic profiles of zebrafish gills in response to zinc supplementation
Source: BMC Genomics. 2010 Oct 11;11:553. doi: 10.1186/1471-2164-11-553 (PMC3091702; doi:10.1186/1471-2164-11-553)
Supplement: Additional file 2 — Interactive Direct Interaction Network representing the molecular interactions between zinc, copper, iron, calcium and proteins encoded by transcripts changed by zinc supplementation. Mini web-site containing index.html and hyperlinked pages in subdirectory describing a Direct Interaction Network automatically generated based on curated interactions contained within the proprietary PathwayArchitect database. Ovals represent proteins and the circles symbolize metal ions. Objects are coloured by their abundance in zebrafish at the time-point they were significantly different from the control is a scale from -4 fold (dark green) to +4 fold (dark red). Where significant differences were found at more than one time-point, the colour overlay shows expression at the first instance. Dark blue squares denote 'binding', and light blue squares 'expression'; green squares stand for 'regulation', green diamonds for 'metabolism', and green circles for 'promoter binding'. Arrow heads indicate directionality of the interaction where annotated. All nodes and edges can be further interrogated by selecting the relative area of the image. [file 1471-2164-11-553-S2.zip › PathwayArchitect Zn xs DIN/1587363.html]

# BINDING:

|  |  |
| --- | --- |
| Type | BINDING |
| Effect | None |


---

|  |  |
| --- | --- |
| Score | 0 |


---

|  |  |
| --- | --- |
| Reference Count | 1 |


---

|  |  |
| --- | --- |
| Mechanism | Unknown |


---

|  |  |
| --- | --- |
| Reference:0 || PMID | 15862967 |
| SourceID | 295751 |
| Species | Human |
| Experimental Condition | in-vivo |
| Description | An interaction between SHBG and MPDU1 was demonstrated by yeast two hybrid screen. Full length SHBG which includes the 29 amino acids amino-terminal signal peptide, bait, fused to the GAL4 DNA binding domain (DBD) and a human prostate cDNA library which included MPDU1, prey, fused to the GAL4 activation domain (AD) were co-transformed into yeast strain AH109. Interactions were detected by growth on media lacking histidine and adenine and by alpha-galactosidase activity. Interacting proteins were identified by DNA purification and sequencing of positive clones. Table 2. |
| Detection Method | two-hybrid-test |
| Source | BIND |
  |


---

|  |  |
| --- | --- |
